# Flow measurement in high-temperature liquid metals and molten salts: principles, recent advances, and deployment challenges

**DOI:** 10.3389/fchem.2026.1977068

**Published:** 2026-10-08

**Authors:** Geon Her, Yeongo Oh, Hyungmo Kim, Seonghyeon Kim

**Affiliations:** 1 Department of Electrical Engineering, Gyeongsang National University, Jinju, Republic of Korea; 2 Bumsung Co., Ltd., Changwon, Republic of Korea; 3 School of Mechanical Engineering, Gyeongsang National University, Jinju, Republic of Korea

**Keywords:** high-temperature flow measurement, liquid metal, molten salt reactor, self-validation, sodium-cooled fast reactor

## Abstract

Next-generation energy systems—including sodium- and lead-cooled fast reactors, molten salt reactors, concentrating solar power, and fusion breeding blankets—rely on high-temperature liquid metals and molten salts operating at approximately 400 °C–700 °C, conditions under which conventional flow instrumentation becomes increasingly constrained. This Mini Review examines electromagnetic, ultrasonic, optical, differential-pressure, vortex, Coriolis, and thermal approaches to flow measurement, with an emphasis on the temperature-sensitive physical properties and sensor–fluid interfaces that govern measurement validity and practical deployment. Differences of several orders of magnitude in electrical conductivity between liquid metals and molten salts favor electromagnetic techniques in liquid metals, particularly sodium, while reducing the signal margin of conductivity-dependent inductive and Lorentz-force approaches in molten salts. Ultrasonic techniques are independent of electrical conductivity and have been demonstrated in high-temperature fluoride-salt loops, although acoustic coupling, thermal-interface stability, and long-term qualification remain important limitations. Across all principles, the central challenge is maintaining measurement validity in the presence of thermal drift, corrosion, changes in wetting, material degradation, and limited opportunities for recalibration. Future high-temperature flow instrumentation will therefore require not only thermally robust sensors but also measurement systems that incorporate condition diagnostics, reduced calibration dependence, and cross-validation between independent physical principles.

## 1 Introduction

Flow measurement in high-temperature liquid metals and molten salts is a major instrumentation challenge in advanced energy systems. Sodium- and lead-cooled fast reactors use liquid metals (Sureshkumar et al., 2013; Schulenberg and Stieglitz, 2010; Li and Yao, 2023), whereas molten salt reactors (MSRs) employ fluoride or chloride salts as coolant or fuel media (Holcomb et al., 2018; Jenet et al., 2025). Concentrating solar power (CSP) systems use nitrate salts (Gill et al., 2013), while sodium is also being investigated for higher-temperature CSP applications (Onea et al., 2024). Lead–lithium breeder loops (Cioli Puviani et al., 2024) and molten-aluminum and glass processing (Kolesnikov et al., 2011; Ihara et al., 2015) provide additional applications. Reliable flow measurement supports heat-balance control, component protection, process monitoring, and nuclear safety functions, yet long service intervals and restricted access can limit *in-situ* recalibration (Schulenberg and Stieglitz, 2010; Holcomb et al., 2018).

Flowmeter capability depends on operating temperature, sensing principle, construction, and packaging. Many commercial instruments are rated below temperatures envisioned for advanced molten salt systems (Baker, 2016; Bai et al., 2024). Elevated temperature degrades piezoelectric elements and bonding layers (Kazys and Vaskeliene, 2021; Bilgunde and Bond, 2015) and progressively demagnetizes permanent magnets (Wang and Zhu, 2021). Thermal gradients and fluid properties perturb ultrasonic propagation (Willatzen, 2003) and conductivity-dependent electromagnetic signals (Priede et al., 2011), while thermal expansion changes calibrated geometries. Corrosion, wetting, fouling, and interfacial degradation further affect long-term stability (Schulenberg and Stieglitz, 2010; Holcomb et al., 2018; Baker, 2016). Radiation adds a reactor-specific qualification requirement whose significance depends on sensing element, packaging, and core proximity (Kazys and Vaskeliene, 2021; Kumar et al., 2021; Zhao et al., 2023).

Fluid properties also determine practical sensing principles. Liquid metals typically have electrical conductivities near 10^6^ S/m, enabling strong electromagnetic coupling and both contact and contactless configurations. Molten salts are several orders of magnitude less conductive, and experimental studies show that signal levels can become insufficient for conductivity-dependent electromagnetic approaches (Kruizenga and Crocker, 2012). This limitation is most relevant to inductive and Lorentz-force techniques, not all electromagnetic flowmeters. Molten salts also impose demanding requirements for material compatibility and sensor interfaces, with limited optical access presenting an additional constraint in high-temperature systems (Holcomb et al., 2018; Arora et al., 2021).

Foundational reviews cover velocity and flow measurement in liquid and heavy liquid metals (Schulenberg and Stieglitz, 2010; Eckert et al., 2007; Eckert et al., 2011; Ratajczak et al., 2017), broader frameworks address MSR instrumentation (Holcomb et al., 2018), and technology-specific reviews examine high-temperature ultrasonics (Kazys and Vaskeliene, 2021). Unlike previous reviews focused on individual fluids or specific technologies, this Mini Review compares sodium, heavy liquid metals, and nitrate, fluoride, and chloride salts using common physicochemical and interfacial criteria, representative performance data, and defined deployment categories. The review also examines sensor hardening, standoff or contactless designs, and cross-validation strategies for maintaining measurement validity as degradation progresses.

## 2 Measurement principles at high temperature

### 2.1 Electromagnetic flowmeters

Electromagnetic flowmeters use motion-induced electromotive force, induced eddy currents, or Lorentz forces in conducting fluids. In sodium systems, permanent-magnet flowmeters (PMFs) provide circuit-level measurements, whereas immersed eddy-current flowmeters (ECFMs), with an excitation coil and two receiving coils, provide local velocity measurements in constrained locations (Sureshkumar et al., 2013; Cui et al., 2025). External electromagnetic frequency flowmeters (EMFMs) have also been tested in lead–bismuth loops (Schulenberg and Stieglitz, 2010). Their lack of moving parts, rapid response, and compatibility with opaque fluids are major advantages. ECFM electronics with millisecond response have been developed for loss-of-flow monitoring (Poornapushpakala et al., 2014).

Temperature effects differ among electromagnetic flowmeters according to the sensing principle. In conventional PMFs, ideal motional electromotive force depends primarily on velocity, magnetic field, and geometry rather than being directly proportional to conductivity. In inductive and Lorentz-force configurations, conductivity changes alter electromagnetic coupling and signal magnitude or phase. In GaInSn, temperature-dependent conductivity shifts the phase response of contactless electromagnetic meters (Priede et al., 2011). Magnetic sources impose an additional thermal limitation because 2:17-type Sm–Co magnets demagnetize at elevated temperatures, although optimized compositions extend their usable range (Wang and Zhu, 2021). One neutron irradiation study reported comparatively small changes in Sm_2_Co_17_ magnetic properties (Zhao et al., 2023). Wetted or electrode-based configurations also face corrosion, wetting instability, and thermoelectric offsets, particularly in heavy liquid metals (Schulenberg and Stieglitz, 2010).

Recent work therefore targets drift, contact limitations, and calibration. Phase-shift designs use relative phase information to reduce sensitivity to conductivity variation (Priede et al., 2011; Looney and Priede, 2019a), while time-of-flight Lorentz-force velocimetry has been proposed to reduce conductivity dependence (Viré et al., 2010). ECFM methods that estimate velocity and conductivity simultaneously separate the measurand from a major drift variable (Krauter and Stefani, 2023). Transient eddy-current flow metering (TECFM) derives velocity from advection of a transient eddy-current pattern and is calibration-free in principle (Forbriger and Stefani, 2015; Krauter and Stefani, 2017; Looney and Priede, 2019b). Fully contactless Lorentz-force velocimetry (LFV) has advanced from theory (Thess et al., 2007) to plant-scale liquid-aluminum trials (Kolesnikov et al., 2011) and dynamic online calibration in metallurgical service (Zheng et al., 2024). These advances strengthen measurement in highly conducting fluids without eliminating the signal-amplitude penalty in low-conductivity molten salts (Kruizenga and Crocker, 2012).

### 2.2 Ultrasonic flowmeters

Ultrasonic flowmeters infer velocity from transit-time differences or Doppler shifts without relying on electrical conductivity. Ultrasonic Doppler velocimetry (UDV) is widely used to obtain velocity profiles in opaque liquid metals (Eckert et al., 2011; Eckert and Gerbeth, 2002), while transit-time systems can use clamp-on or standoff configurations (Baker, 2016). Among the methods reviewed, ultrasonics offers the broadest demonstrated conductivity-independent route to non-intrusive measurement across both fluid classes.

High-temperature limitations span the signal chain: conventional PZT elements can degrade, bond layers can fail below nominal Curie temperature, and couplants may dry, creep, or delaminate (Kazys and Vaskeliene, 2021; Bilgunde and Bond, 2015). Temperature gradients change sound speed and refract the beam. Although uniform sound-speed effects may cancel in ideal transit-time formulations, nonuniform fields and path distortion still cause error (Willatzen, 2003; Wang et al., 2004). Corrosion and changes in wetting at interfaces or waveguide terminations alter acoustic transmission and can destabilize the measurement path (Schulenberg and Stieglitz, 2010; Kazys et al., 2005).

Piezoelectrics with high Curie temperatures and standoff structures mitigate these constraints. LiNbO_3_ transducers produced signals after 300 h in lead–bismuth at 350 °C, and separate gamma irradiation tests showed no noticeable changes in pulse response or transfer coefficient at 22 MGy (Kazys et al., 2005). These component tests do not establish performance under combined thermal and radiation exposure or continuous flowmeter accuracy. GaPO_4_ guided-wave transducers were demonstrated on piping near 600 °C (Dhutti et al., 2019). Waveguides and buffer rods move sensitive components away from the hot zone, as demonstrated by UDV measurements in molten CuSn at 620 °C (Eckert et al., 2003). In FLiNaK, transit-time ultrasonics was cross-validated against pressure-drop measurements at 600 °C (Arora et al., 2021), and later experiments used commercial high-temperature ultrasonic meters (Chen Y. S. et al., 2024). Signal loss caused by an air gap in the acoustic path was reported during FLUSTFA operation (Zhang et al., 2024). These studies establish feasibility in fluoride salt loops, with coupling and thermal-interface stability remaining constraints on service life.

### 2.3 Optical and fiber-optic sensing

Fiber-optic sensing offers electromagnetic immunity, multiplexing, and potentially high radiation tolerance (Kumar et al., 2021; Laffont et al., 2018). Limited optical access and the opacity of liquid metals restrict direct optical velocimetry, so fiber-based flow sensors usually transduce flow into a thermal or mechanical response. A regenerated fiber Bragg grating (FBG) hot-wire sensor, for example, measured gas velocities of 0.066–0.66 m/s from room temperature to 800 °C using flow-induced heat transfer from an optically heated fiber (Chen et al., 2014). These results demonstrate high-temperature capability in gas rather than operation in liquid metals or molten salts.

The maturity of grating technology is supported by evaluations of regenerated silica gratings for long-duration service near 900 °C (Kumar et al., 2021) and sapphire FBGs at substantially higher temperatures (Habisreuther et al., 2015). Distributed fiber-optic temperature sensing has also operated in a molten-salt loop (Arora et al., 2021), supporting auxiliary thermal diagnostics. The transduction and packaging interface remains the main barrier to primary flow metering. Practical devices must maintain a stable relationship between flow and response while protecting fibers, coatings, feedthroughs, and wetted elements against thermal cycling and corrosion. Long-duration demonstrations of packaged fiber-optic flowmeters in liquid metals or molten salts remain limited.

### 2.4 Differential-pressure and vortex meters

Differential-pressure (DP) devices infer flow from calibrated pressure loss across an orifice, Venturi, or other restriction, with conversion coefficients that depend on geometry and fluid properties. Thermal expansion changes throat areas and pressure-tap geometry. Temperature-dependent density affects conversion from pressure drop to flow, whereas viscosity influences calibration through changes in Reynolds number. In molten salts, impulse lines and taps can freeze or plug below the salt melting range, making trace heating and layout integral to the system (Gill et al., 2013; Baker, 2016). DP measurement remains useful, particularly as a reference, provided that adequate thermal management is maintained.

Vortex meters infer flow from vortex-shedding frequency behind a bluff body and avoid impulse lines, but calibration depends on bluff-body geometry and Reynolds number. Thermal expansion changes the characteristic dimension, and changes in viscosity can shift operation outside the constant-K-factor regime. Designs for operation at low Reynolds numbers have been studied to extend linearity (Končar et al., 2022). Flow inferred from valve pressure drops at MSTL was insufficiently reliable in nitrate salt (Gill et al., 2013). A separate nitrate-salt Venturi was calibrated in the target salt against a vortex meter, with greater uncertainty at low Reynolds numbers (Aakre, 2021). FLiNaK pressure-drop measurements supported cross-validation (Arora et al., 2021), whereas chloride-salt Venturi tests showed substantial scatter and corrosion of wetted components (Bai et al., 2024). Vortex meters have documented use in heavy-liquid-metal facilities (Cioli Puviani et al., 2024) and a nitrate salt test platform (Kraft et al., 2025).

### 2.5 Coriolis and thermal meters

Coriolis meters measure mass flow directly from vibrating-tube inertia, which is attractive when density varies strongly with temperature. At high temperatures, their limitations are mainly structural rather than related to conductivity. Temperature-dependent changes in elastic modulus alter tube dynamics and can produce zero and span drift, effects that can be reduced through model-based correction (Costa et al., 2020). Drive and pickoff assemblies, attachments, and electronics also impose temperature ceilings (Baker, 2016). Coriolis meters have been used in heavy-liquid-metal facilities (Cioli Puviani et al., 2024) and in sodium comparison measurements at 240 °C–340 °C (Aakre et al., 2023), although reference accuracy requires validation for the specific fluid and operating conditions.

Thermal meters infer mass flow from imposed heat and convective transport. Despite their mechanical simplicity, their response depends on thermal conductivity, heat capacity, flow regime, and surface condition. Corrosion or deposits can therefore alter the calibrated heat-transfer path. A dedicated thermal mass flowmeter has been experimentally demonstrated for heavy liquid metals (Cioli Puviani et al., 2024). Thermal-wave time-of-flight metering has been proposed for molten salts, although the published proof of concept used a water loop (Škarohlíd et al., 2025). Demonstration of the principle does not establish material compatibility or corrosion resistance at high temperature.

## 3 Deployment status by fluid class

Deployment maturity varies across fluid classes, ranging from operational service to loop-scale demonstration or early development. Figure 1 summarizes deployment evidence, and Table 1 links sensing principles to high-temperature dependencies, failure mechanisms, and representative quantitative demonstrations. Existing reviews guided targeted searches across fluid classes and sensing principles, including primary studies, public laboratory reports, and a doctoral dissertation identified through reference tracing. The survey sought representative demonstrations rather than exhaustive coverage. Operational service denotes documented use in an operating reactor or industrial process beyond a dedicated sensor trial. Loop-scale demonstration/use requires actual flow or velocity measurement in the target fluid in an experimental facility. Component tests, auxiliary sensing, tests in substitute fluids, and unsuccessful flow estimates do not independently establish deployment. Open cells indicate no qualifying example in the reviewed literature, not technical infeasibility. Each cell records the strongest identified evidence for a configuration within that fluid class; the categories do not rank accuracy or lifetime.

**FIGURE 1:**
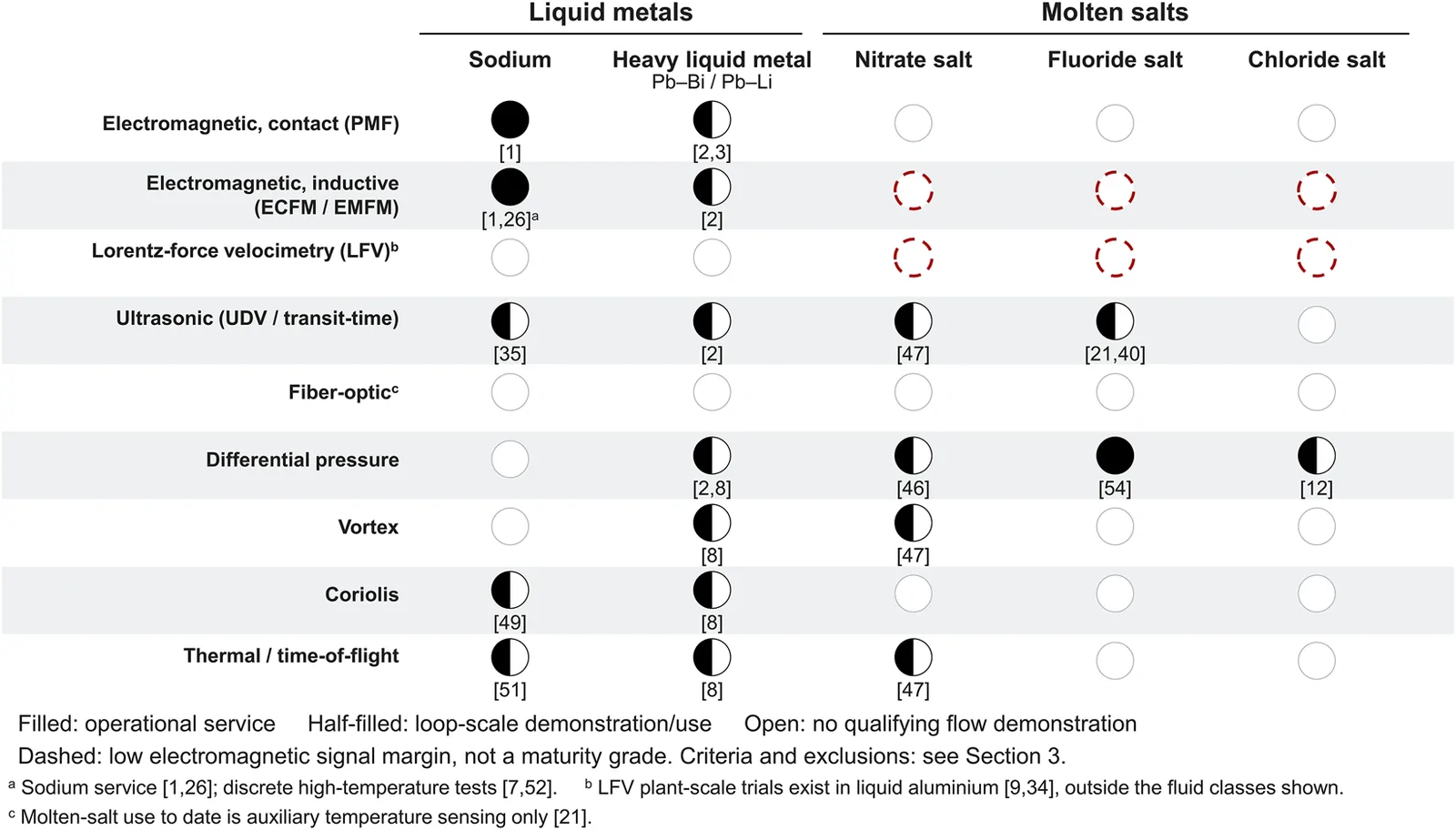
Deployment maturity of flow measurement technologies in high-temperature liquid metals and molten salts. Filled, half-filled, and open cells indicate operational service, loop-scale demonstration/use, and no representative flow demonstration identified, respectively. Supporting references are shown below populated cells. Dashed outlines denote low electromagnetic signal margin, not a maturity category. Classification criteria and exclusions are defined in Section 3. Bracketed numbers refer to: [1] Sureshkumar et al., 2013; [2] Schulenberg and Stieglitz, 2010; [3] Li and Yao, 2023; [7] Onea et al., 2024; [8] Cioli Puviani et al., 2024; [9] Kolesnikov et al., 2011; [12] Bai et al., 2024; [21] Arora et al., 2021; [26] Poornapushpakala et al., 2014; [34] Zheng et al., 2024; [35] Eckert and Gerbeth, 2002; [40] Chen Y. S. et al., 2024; [46] Aakre, 2021; [47] Kraft et al., 2025; [49] Aakre et al., 2023; [51] Reimche et al., 1985; [52] Krauter et al., 2023; [54] Guymon, 1973.

**TABLE 1:** Flow measurement principles for high-temperature liquid-metal and molten-salt systems: dominant high-temperature dependencies, strengths, failure mechanisms, representative research responses, and representative demonstrations with reported operating/performance data.

Principle (dominant high-T dependency)	High-T strengths/interface	Dominant failure mechanisms	Representative research responses	Representative demonstrations/reported data
Electromagnetic, contact (PMF) (field/electrodes/interface)	• Fast response • Established sodium service • Wetted flow tube; external magnet	• Magnet demagnetization (Wang and Zhu, 2021) • Electrode corrosion, wetting, thermoelectric drift (Schulenberg and Stieglitz, 2010)	• High-temperature magnets (Wang and Zhu, 2021) • Sm_2_Co_17_ irradiation evaluation (Zhao et al., 2023)	• FBR circuit service (Sureshkumar et al., 2013) • KALLA LBE: 2%–4% accuracy after calibration and correction (Schulenberg and Stieglitz, 2010)
Electromagnetic, inductive (ECFM/EMFM) (electromagnetic coupling/conductivity)	• ECFM: immersed local monitoring • ms-class response (Poornapushpakala et al., 2014) • ECFM: wetted casing; external EMFM: no wetted sensor (Schulenberg and Stieglitz, 2010)	• Coil thermal limits • Ni-coating Curie transition: unreliable readings at 330 °C–360 °C in the tested ECFM (Krauter et al., 2023) • Conductivity-dependent response • Calibration burden (Priede et al., 2011; Forbriger and Stefani, 2015)	• TECFM (Forbriger and Stefani, 2015; Krauter and Stefani, 2017; Looney and Priede, 2019b) • Phase-shift designs (Priede et al., 2011; Looney and Priede, 2019a) • Simultaneous conductivity estimation (Krauter and Stefani, 2023)	• Sodium: Phénix/FBTR service (Sureshkumar et al., 2013; Poornapushpakala et al., 2014); discrete tests up to 700 °C over 2 days (Onea et al., 2024; Krauter et al., 2023) • Pb–Bi: external EMFM flow tests at 200 °C (Schulenberg and Stieglitz, 2010)
Lorentz-force velocimetry (LFV) (conductivity/magnetic force)	• Fully contactless	• Low signal in weak conductors (Kruizenga and Crocker, 2012)	• Time-of-flight approach (Viré et al., 2010) • Online dynamic calibration (Zheng et al., 2024)	• Liquid-Al plant trials: 780 °C; estimated relative flow error 2.34%; 1,119 s calibration run (Kolesnikov et al., 2011) • Metallurgical online measurement (Zheng et al., 2024)
Ultrasonic (UDV/transit-time) (acoustic path/transducer condition)	• Independent of conductivity • Clamp-on: no wetted sensor; standoff: depends on design • Velocity profiles with UDV (Eckert et al., 2011; Eckert and Gerbeth, 2002)	• PZT/bond degradation (Kazys and Vaskeliene, 2021; Bilgunde and Bond, 2015) • Thermal-gradient path distortion (Willatzen, 2003) • Wetted-end corrosion (Schulenberg and Stieglitz, 2010) • Acoustic-path/coupling loss (Zhang et al., 2024)	• LiNbO_3_ and GaPO_4_ (Kazys and Vaskeliene, 2021; Kazys et al., 2005; Dhutti et al., 2019) • Standoff waveguides (Kazys et al., 2005; Eckert et al., 2003; Jen et al., 2000; Liao et al., 2020)	• Sodium UDV (Eckert and Gerbeth, 2002); nitrate salt platform use (Kraft et al., 2025) • Heavy liquid metal UDV (Schulenberg and Stieglitz, 2010) • LiNbO_3_ component tests: Pb–Bi 350 °C, 300 h; separate gamma test at 22 MGy (Kazys et al., 2005) • FLiNaK 600 °C, subsequent loop use (Arora et al., 2021; Chen Y. S. et al., 2024)
Fiber-optic (grating/thermal readout/packaging)	• EM immunity • Multiplexing • Radiation tolerance (Kumar et al., 2021; Laffont et al., 2018) • Interface depends on packaging	• Thermal-readout dependence • Wetted packaging and corrosion protection unresolved	• Regenerated FBGs (Kumar et al., 2021; Chen et al., 2014) • Sapphire FBGs (Habisreuther et al., 2015)	• Molten-salt auxiliary temperature sensing only (Arora et al., 2021) • Gas flow demonstration: 0.066–0.66 m/s, up to 800 °C (Chen et al., 2014); not evidence in target liquids
Differential pressure (geometry/fluid properties)	• Mature infrastructure • Reference-meter role • Wetted restriction and pressure taps	• Thermal expansion • Fluid-property dependence • Impulse-line freezing/plugging (Gill et al., 2013; Baker, 2016)	• Heated impulse lines (Gill et al., 2013) • Customized Venturis (Bai et al., 2024)	• Nitrate Venturi calibration (Aakre, 2021); FLiNaK DP (Arora et al., 2021); chloride 500 °C, max. SD 9.71 g/s (Bai et al., 2024) • Heavy liquid metal campaigns (Schulenberg and Stieglitz, 2010; Cioli Puviani et al., 2024) • MSRE coolant-salt Venturi service (Guymon, 1973)
Vortex (bluff-body geometry/Reynolds number)	• Industrial maturity • Intrusive, wetted bluff body	• Thermal expansion • Low-Re K-factor nonlinearity (Končar et al., 2022)	• Low-Re bluff-body redesign (Končar et al., 2022)	• Heavy liquid metal facility use (Cioli Puviani et al., 2024) • Nitrate platform use when all flow is drawn from cold storage (Kraft et al., 2025)
Coriolis (tube dynamics/elastic modulus)	• Direct mass flow • Reference accuracy needs validation (Cioli Puviani et al., 2024) • Wetted vibrating tube	• Modulus-temperature drift (Costa et al., 2020) • Drive/pickoff thermal limits (Baker, 2016)	• Model-based temperature correction (Costa et al., 2020)	• IELLLO PbLi measurements; test meter near its density measurement limit (∼10,000 kg/m³), reference accuracy unverified (Cioli Puviani et al., 2024) • Sodium: Coriolis comparison at 240 °C–340 °C (Aakre et al., 2023)
Thermal/time-of-flight (transport properties/surface condition)	• Simple structure • Heated section or existing thermometry; contact depends on configuration	• Fluid-property drift • Surface fouling/corrosion	• Dedicated heavy liquid metal meter (Cioli Puviani et al., 2024) • Thermal-wave concept (Škarohlíd et al., 2025) • System-level thermal time-of-flight (Kraft et al., 2025; Kraft et al., 2024)	• Heavy liquid metal loops (Cioli Puviani et al., 2024) • Sodium thermocouple correlation: 1–10 m/s (Reimche et al., 1985) • Water-loop proof of concept (Škarohlíd et al., 2025); thermal oil HTF data (Kraft et al., 2024) • EMSP molten-salt (Kraft et al., 2025): inlet 300 °C–440 °C, 5.8–12.1 kg/s, mean reference deviation <5% after linear calibration, >100 experiments

Reported performance values are specific to the cited studies and should not be interpreted as directly comparable uncertainty or lifetime metrics.

### 3.1 Sodium: mature electromagnetic instrumentation

Sodium has the most mature high-temperature flow instrumentation among the systems considered here. PMFs provide primary- and secondary-circuit flow measurements, while ECFMs support local monitoring, including fuel-assembly outlet measurements, with operating experience in systems such as FBTR and Phénix (Sureshkumar et al., 2013; Poornapushpakala et al., 2014). Complementary measurements include UDV for thermal-hydraulic experiments (Eckert and Gerbeth, 2002) and thermocouple correlation velocimetry in liquid sodium (Reimche et al., 1985). Current development emphasizes temperature capability, reduced calibration requirements, and long-term validation rather than basic measurement feasibility. High-temperature immersed ECFMs have been tested in sodium and investigated in the 1000 K SOLTEC-2 loop for SFR and CSP applications (Onea et al., 2024; Krauter et al., 2023). Drift and degradation during extended service remain important qualification issues.

### 3.2 Heavy liquid metals: multiple candidates without a single dominant standard

Lead–bismuth and lead–lithium remain sufficiently conductive for electromagnetic measurement, making the signal-strength constraints encountered in molten salts less severe. The main challenge is maintaining magnetic, geometric, and interfacial stability in corrosive high-temperature liquid metal. Permanent-magnet electromagnetic flowmeters in the KALLA lead–bismuth program achieved a reported accuracy of 2%–4% after appropriate calibration and correction (Schulenberg and Stieglitz, 2010). Temperature can nevertheless alter both magnetic field and flow distribution (Li and Yao, 2023). Corrosion and wetting further complicate electrode-based designs and calibration transfer between facilities.

European facilities use vortex and Coriolis meters alongside electromagnetic devices (Cioli Puviani et al., 2024), and Pb–Li programs have evaluated multiple electromagnetic configurations. A dedicated thermal mass flowmeter was also developed and tested for heavy-liquid-metal service (Cioli Puviani et al., 2024). The range of viable principles is broader than for molten salts, with less standardization than in sodium systems. Qualification thus centers on long-term interface stability, temperature-dependent calibration, and facility integration rather than basic sensing feasibility.

### 3.3 Molten salts: chemistry-dependent deployment constraints

Instrumentation maturity varies with molten salt chemistry and operating temperature. Lower electrical conductivity reduces signal margin for inductive and Lorentz-force electromagnetic techniques (Kruizenga and Crocker, 2012), while contact-type designs also require corrosion resistance, stable electrode interfaces, and compatible materials (Holcomb et al., 2018). Ultrasonic and vortex measurements have been demonstrated in nitrate salts at the Évora Molten Salt Platform (Kraft et al., 2025). The Solar Salt receiver’s 600 °C limit is a system design value rather than a demonstrated flowmeter capability (Frantz et al., 2023). Unlike the unreliable flow estimates obtained from valve pressure drops at MSTL (Gill et al., 2013), calibration of a Venturi in nitrate salt provides direct evidence of DP flow measurement (Aakre, 2021).

Fluoride salts used in MSR-related systems generally operate at higher temperatures and impose more demanding material and interface requirements. Historical operating experience includes a Venturi in the MSRE coolant salt loop (Guymon, 1973). Transit-time ultrasonics was cross-validated against pressure-drop measurements at 600 °C in FLiNaK (Arora et al., 2021), and later experiments used high-temperature ultrasonic meters for loop flow measurement (Chen Y. S. et al., 2024). Long-duration qualification remains limited, and FLUSTFA signal loss highlights the importance of acoustic coupling and installation stability (Zhang et al., 2024).

Customized Venturi meters have operated in chloride-salt loops under high-temperature, corrosive conditions, although scatter in the flow measurements and degradation of wetted loop components were reported (Bai et al., 2024). Published demonstrations therefore remain dominated by custom pressure-based approaches rather than broadly qualified non-intrusive flowmeters.

Molten-salt deployment constraints depend on salt chemistry and system configuration. Nitrate salts have ultrasonic and vortex demonstrations; fluoride salts combine historical DP service with recent ultrasonic measurement but limited lifetime qualification; and chloride salts still rely largely on customized solutions.

## 4 Discussion and outlook

### 4.1 Synthesis: dominant dependencies of measurement validity

Contact electromagnetic meters depend on magnetic-field stability, geometry, electrodes, and sensor–fluid interfaces; inductive and Lorentz-force variants depend more strongly on conductivity and electromagnetic coupling. Ultrasonic meters depend on acoustic-path stability, transducer condition, coupling, and temperature-dependent propagation. DP meters depend on calibrated geometry and fluid properties, vortex meters on bluff-body geometry and hydrodynamic similarity, Coriolis meters on structural properties, and thermal meters on heat transfer.

These dependencies help explain differences in deployment maturity, with high liquid-metal conductivity supporting strong electromagnetic coupling and several contactless approaches. Molten salts provide lower signal margins for conductivity-dependent techniques and add uncertainties in thermophysical properties, corrosion, and long-term interface stability (Holcomb et al., 2018; Kruizenga and Crocker, 2012). Successful liquid-metal operation therefore does not establish transferability to fluoride or chloride salts.

### 4.2 Sensor hardening versus standoff and contactless design

Two engineering strategies recur across the reviewed technologies. Sensor hardening keeps the transduction element near the measurement region while increasing temperature capability through piezoelectrics with high Curie temperatures (Kazys and Vaskeliene, 2021; Griffin et al., 2009), thermally stable bonding, high-temperature permanent magnets (Wang and Zhu, 2021), and heat-tolerant electronics. This approach preserves strong direct coupling, although service life remains tied to the materials in the hot zone. A high-temperature piezoelectric element may, for example, remain functional after the bond layer or couplant begins to degrade (Kazys and Vaskeliene, 2021; Bilgunde and Bond, 2015).

Standoff and contactless designs instead move sensitive components away from the fluid or eliminate wetted interfaces. Acoustic buffer rods and waveguides protect transducers but add attenuation, temperature-dependent transfer functions, and installation sensitivity (Kazys et al., 2005; Eckert et al., 2003; Jen et al., 2000; Liao et al., 2020). External LFV eliminates electrodes, while transient or phase-based eddy-current methods reduce some calibration and contact limitations (Kolesnikov et al., 2011; Priede et al., 2011; Forbriger and Stefani, 2015; Thess et al., 2007). Neither strategy is universally superior: hardening favors strong local coupling, whereas standoff or contactless sensing is attractive when corrosion and thermal exposure dominate. Selection depends on fluid class, geometry, allowable pressure loss, signal margin, and tolerable uncertainty over the service interval.

### 4.3 From individual meters to degradation-aware measurement systems

Long-duration reactor service at high temperature requires delaying degradation and detecting loss of measurement validity. Redundancy across sensing principles combines sensors with different failure mechanisms. FLiNaK experiments cross-validated ultrasonics against pressure-drop measurement (Arora et al., 2021), while heavy-liquid-metal facilities have used multiple principles (Schulenberg and Stieglitz, 2010; Cioli Puviani et al., 2024). In systems designed for cross-validation, disagreement between independent principles can serve as a diagnostic indicator rather than a retrospective observation.

A complementary approach combines self-diagnosis with reduced calibration dependence. Self-validating sensor concepts provide a framework for reporting measurement status and confidence (Henry and Clarke, 1993; Henry et al., 2000; Chen et al., 2025). TECFM reduces conventional calibration dependence (Forbriger and Stefani, 2015; Krauter and Stefani, 2017), simultaneous conductivity estimation separates an electromagnetic drift variable from velocity (Krauter and Stefani, 2023), and ultrasonic echo quality can indicate coupling condition. Machine learning has been explored for Coriolis error correction and prediction of in-use ultrasonic flowmeter deviations in other environments (Liu et al., 2001; Li et al., 2023). Application of machine learning to validation and error correction in high-temperature liquid-metal or molten-salt flowmeters remains to be demonstrated. Data-driven methods are therefore best treated as supplements to physics-based calibration for integrating diagnostics, detecting drift, and quantifying confidence when direct recalibration is unavailable.

### 4.4 Emerging measurement principles

Thermal-wave metering is being developed for molten-salt service, although published validation used a water loop (Škarohlíd et al., 2025). At the system level, thermal time-of-flight methods use natural or induced temperature transients to estimate mass-flow distribution without a dedicated wetted sensor. At the Andasol III CSP plant using a thermal-oil heat-transfer fluid (HTF), 94% of analyzed operational data fell within ±5% of full-scale flow (Kraft et al., 2024). The method was subsequently demonstrated at the Évora Molten Salt Platform using collector defocusing to generate thermal transients (Kraft et al., 2025). A nuclear magnetic resonance flowmeter has been proposed for simultaneous monitoring of coolant flow and condition (Davydov et al., 2022). Flow-enhanced electrochemical sensing in molten-salt safeguards testbeds, although not a primary flowmeter, shows how flow-dependent responses can be coupled with fluid-state information (Doniger et al., 2024). For liquid metals, contactless inductive tomography and inversion of externally measured magnetic-field perturbations seek cross-sectional velocity information rather than only bulk flow (Eckert et al., 2011; Ratajczak et al., 2017; Chen R. et al., 2024).

### 4.5 Implications for high-temperature deployment

No single principle is universally optimal for high-temperature flow measurement. Sodium shows the maturity achievable when fluid properties favor the sensing physics and operational experience is extensive. Heavy liquid metals retain several viable options but impose stronger material and interface constraints, while molten salts combine lower electromagnetic coupling with demanding corrosion environments, temperature-sensitive properties, and limited recalibration opportunities. Recent molten-salt ultrasonic demonstrations, continued development of contactless electromagnetic methods, and emerging thermal-wave and thermal time-of-flight approaches show substantial progress (Arora et al., 2021; Forbriger and Stefani, 2015; Thess et al., 2007; Zheng et al., 2024; Kraft et al., 2025; Škarohlíd et al., 2025; Frantz et al., 2023; Kraft et al., 2024). The central challenge is not simply measurement at high temperature but maintaining traceable measurement validity throughout extended service. Future systems should combine an appropriate primary measurement principle with thermal and condition diagnostics, reduced calibration dependence, and independent cross-checks that reveal degradation before undetected flow errors develop.
